# Current developments in elastic and acoustic metamaterials science

**DOI:** 10.1098/rsta.2023.0369

**Published:** 2024-09-09

**Authors:** Giuseppe Failla, Alessandro Marzani, Antonio Palermo, Andrea Francesco Russillo, Daniel Colquitt

**Affiliations:** ^1^ Department of Civil, Energy, Environmental and Materials Engineering (DICEAM), University of Reggio Calabria, Via Zehender, Reggio Calabria 89124, Italy; ^2^ Department of Civil, Chemical, Environmental and Materials Engineering (DICAM), University of Bologna, Viale del Risorgimento 2, Bologna 40136, Italy; ^3^ Department of Mathematical Sciences, University of Liverpool, Liverpool L69 7ZL, UK

**Keywords:** metamaterial, elastodynamics, acoustics, design, computational modelling, wave control

## Abstract

The concept of metamaterial recently emerged as a new frontier of scientific research, encompassing physics, materials science and engineering. In a broad sense, a metamaterial indicates an engineered material with exotic properties not found in nature, obtained by appropriate architecture either at macro-scale or at micro-/nano-scales. The architecture of metamaterials can be tailored to open unforeseen opportunities for mechanical and acoustic applications, as demonstrated by an impressive and increasing number of studies. Building on this knowledge, this theme issue aims to gather cutting-edge theoretical, computational and experimental studies on elastic and acoustic metamaterials, with the purpose of offering a wide perspective on recent achievements and future challenges.

This article is part of the theme issue ‘Current developments in elastic and acoustic metamaterials science (Part 1)’.

## 1. Introduction

Despite being a relatively new research area in materials science, metamaterials already possess a solid theoretical, computational and experimental foundation, showcasing their potential for engineering applications. Originally introduced in the field of electromagnetism, the term ‘metamaterial’ now indicates, in a broader sense, an engineered material whose effective properties arise from a tailored design of its architecture, either at the macro-scale or at the micro-/nano-scales. Indeed, recent advances in fabrication techniques now allow macro-, micro-, as well as nano-scale metamaterials to be conceived with any architecture, including lattice and composite ones. The terms elastic and acoustic refer to metamaterials devised for applications in elastostatics, elastodynamics and acoustics. Examples include metamaterials with high stiffness to resist deformation, high strength to prevent permanent deformation, high toughness for energy absorption without cracking, negative stiffness for energy absorption, negative Poisson’s ratio for indentation protection and reduced surface wrinkling, negative compressibility for design of actuators or force amplifiers, structural anisotropy to guide loading into preferred directions or features. In the dynamic regime, the main applications include the attenuation of mechanical vibration and/or sound at specific frequency ranges, i.e. band gaps, thanks to periodicity or local resonance effects induced by resonators. Additional examples include cloaks that can generate hidden regions from elastic or acoustic waves, zero-stiffness metamaterials that can mitigate vibrations, origami-based metamaterials that can realize the directional transmission of elastic waves and so on. Currently, a considerable number of theoretical, computational and experimental studies substantiates the remarkable properties of metamaterials, with several review papers and books [1–8].

Research on metamaterials is now targeting new challenges. A primary target is to devise metamaterials with enhanced properties or innovative, exotic ones for engineering applications. Moreover, there is a strong and growing interest in developing metamaterials with adaptable properties that may respond differently at different environmental conditions. A companion emerging frontier is to obtain multi-functional metamaterials with properties useful for different purposes. To achieve all these objectives, a crucial role is played by rational design methodologies and fabrication techniques. In this regard, increasing attention is given, for example, to hierarchical or bioinspired designs in order to establish a systematic methodology for generating the architecture of metamaterials. Among fabrication techniques, additive manufacturing methods can fabricate architectures with high resolution, arbitrary complexity and high feature fidelity, even at very small scales. These methods now enable the rapid development of architected metamaterials and drastically reduce the design-to-experimental-validation cycle. In parallel, the development of advanced theoretical and computational models is a fundamental objective to ensure the required accurate description of metamaterial behaviours of increasing complexity.

While an exhaustive description is almost prohibitive, this theme issue will provide a broad perspective on the most recent developments in elastic and acoustic metamaterials science, with 21 papers on novel design methodologies and typologies, modelling and analysis techniques and engineering applications. The theme issue is divided into two parts, both covering the full breadth of these topics. Here follows a description of Part 1, with a general overview of the recent literature in the field.

## 2. Design methodologies and typologies

Research on elastic and acoustic metamaterials devotes a great deal of attention to the development of rational and systematic design criteria. Since the conception of metamaterials, lattices have been natural candidates to achieve high-strength lightweight materials, thanks to their porous structures and tailored material properties based on suitable well-defined unit cell geometries. Using three-dimensional printing techniques, a variety of lattice metamaterials with exceptional and unusual properties were designed and manufactured in the last few decades, using, e.g. polymers, metals and ceramic composites [3,9]. Among others, bio-inspired design strategies are of particular interest for lattice metamaterials [10,11], to obtain, for instance, light-weight and high-strength metamaterials for aerospace applications, lightweight construction, energy absorption [10] or three-dimensional soft network metamaterials with unusual mechanical properties for innovative bio-integrated electronics, biomass probing sensors and devices [11]. In the context of bio-inspired design, hierarchy and structural gradient are effective principles; while hierarchy generates micro-structures at multiple and different length scales, structural gradient drives the spatial distribution of materials. Hierarchy and structural gradient may produce enhanced mechanical and multi-functional properties in bio-inspired lattice metamaterials [12–20]. For example, a soft network lattice metamaterial combining two-dimensional lattice topologies (e.g. triangular, Kagome and honeycomb) typical of cellular materials with stretchable horseshoe/serpentine micro-structures was developed in Jang *et al*. [16] to attain simultaneously large levels of stretchability and a relatively high mechanical strength, for applications in tissue engineering and stretchable bio-integrated electronics; building on this concept, further studies [17] formulated a pertinent theoretical model to study the deformation mechanism and predict key mechanical quantities for design optimization. Inspired by the structure characteristics of the skeletal system of deep-sea glass sponge, a vertex modified body-centred cubic lattice metamaterial made of stainless steel was proposed in Wang *et al.* [18], which outperforms corresponding conventional lattices in terms of deformation stability, energy absorption and strength, with potential applications in aerospace engineering as well as medical implants. On adopting a nacre-like design, block lattice metamaterials with elastic wave filtering and enhanced mechanical properties were developed in Bollineni *et al*. [19], leveraging artificial neural networks for forward prediction, parameter and topology designs. Furthermore, mimicking the crossed-lamellar design of the shell of the *Strombus gigas* mollusc, whose hierarchy consists of four distinct lamellar-shaped features assembled in a three-dimensional arrangement, a lattice metamaterial was designed in Chen *et al*. [20] to circumvent the typical trade-offs between strength-ductility and strength-density; in particular, the hierarchical structural feature proves capable of controlling the evolution of cracks and other types of localized deformation such as shear bands, resulting in improved strength and toughness. In the theme issue, Chirikjian [21] proposes an original group theoretic approach to the design of elastic metamaterials, with a focus on lattice metamaterials. Inspired by the many existing deployable structures in nature and synthetic mechanisms that preserve symmetry as their configurations evolve, the approach delivers elastic metamaterials preserving symmetry while passively deforming. It is demonstrated that group theory can be used as a systematic approach to describe configurational changes, reducing symmetrical deployable metamaterials and metamaterial structures to their fundamental and irreducible components and simplifying the design of very complex structures. Specifically, a theory for generating arrangements/configurations of bodies (or voids) in point contact is developed to produce feasible candidate metamaterial structures.

Among the plethora of lattice designs, a peculiar typology arises in so-called Triply Periodic Minimal Surface (TPMS) lattices. Thanks to their continuous surface geometry with gradual changes in curvature, TPMS structures enjoy an efficient load distribution and stress transfer across the surface; these properties minimize stress concentrations and eventually fatigue damage [22,23], making TPMS structures ideally suitable for biomimetic design. Among others, functional gradation and multi-morphology hybridization were proposed as effective design optimization strategies for TPMS lattices [24]. In the theme issue, the paper by Nooghabi *et al*. [25] proposes a plate-like metastructure built by tessellating a unit three-dimensional symmetric Schwarz primitive cell, which belongs to the family of TPMSs. In particular, the Schwarz primitive cell exhibits significant strength to compression and fracture toughness; these static properties are well known and research is ongoing to design porous and bone-mimicking scaffolds, based on such geometries, for medical applications and bone implants. The plate-like metastructure proposed by Nooghabi *et al*. [25] investigates the potential of the Schwarz primitive cell for elastic wave control and manipulation; specifically, mitigation and focusing of elastic out-of-plane bending waves is achieved by local variations of the geometry of the plate, based on increasing porosity and adding mass. The proposed concepts are corroborated by numerical analysis and experimental validation.

Emerging targets of research on metamaterials include tunability and programmability. Indeed, several applications require adaptive programmable materials capable of maintaining or changing their shapes and properties when subjected to external excitation such as electrical or magnetic fields, light, temperature, moisture, pressure, external load and boundary conditions. Programmable metamaterials can exhibit multiple stable states [26–31] and advanced functionalities obtained by switching between different stable states under a controlling stimulation. For example, buckling-driven metamaterials were proposed to engineer soft mechanisms used in robotics as force switch, kinematic (position/velocity) controllers, pick and place grasping mechanism [30], to develop energy-efficient building skins changing configuration to reflect or transmit light [31]; in these cases, buckling is activated by ad hoc imperfections mimicking the desired actuation mode [30], by external loads or thermal actuation owing to environmental temperature changes [31]. On-demand programmability in lattice metamaterials was obtained by multi-physical mechanics involving external stimuli-like electrical and magnetic fields, light, temperature and shape memory effects [32]. The effective elastic moduli can be actively controlled in piezoelectric lattices as a function of voltage, leading to stiffer or softer behaviour of a single lattice architecture in an on-demand framework as per operational requirements, even after its fabrication [33,34]. A magnetic field can lead to on-demand modulation of effective elastic properties in a contactless framework [35,36]. Programmability of thermal expansion and load-bearing capacity can be attained simultaneously in multi-phase metamaterials containing framework structures [37]. Recently, the concept of pneumatic elastostatics and deployability in elastic metamaterials was proposed based on inflatable lattices that may exhibit extreme specific stiffness along with on-demand tunability [38]. In the theme issue, Kundu *et al*. [39] propose piezoelectric beam lattices where the effect of random multiple disorders and damages of complex shapes, sizes and distributions can be shielded through active cloaks controlled by voltage-dependent modulation of the stress fields within the cloaking region. Mechanical cloaks aim to alter the elastic response around defects or voids making them unrecognizable from their homogeneous surroundings, i.e. cloaking a defect implies making it invisible in terms of homogenized mechanical response. For on-demand cloaking, the authors develop a computational framework involving multi-physical finite element simulations and numerical optimization determining element-wise voltages in the cloaking region, which redistribute stress and strain components to negate the effect of damage in the strain field beyond this region.

For tunability and programmability, promising materials are shape memory polymers (SMPs), i.e. stimulus-responsive smart materials with stiffness tuneability, active deformation and shape memory effects under external stimuli such as heat [40], light [41], electricity [42] and magnetism [43]. SMPs are ideal candidates as four-dimensional printed materials [44–47], i.e. three-dimensional printed materials with changing properties in time. In the theme issue, Wan *et al*. [48] propose three-dimensional multi-stable metamaterials composed of SMP curved beams and support frames, with reconfigurable shapes, tunable mechanical properties and programmable deformation sequences utilizing a four-dimensional printing method. The mechanical properties and deformation of the proposed three-dimensional multi-stable metamaterials are investigated thoroughly under compressive load by finite element analyses and corroborated by experiments, concluding that force-displacement curves and multi-stable deformation sequences can be spontaneously tuned and programmed by controlling the temperature and thickness of the curved beams. Different designs of the three-dimensional units are explored, either for low-energy dissipation and reduced material costs or for high-energy dissipation at low volumes.

## 3. Modelling and analysis

Developing accurate and computationally efficient models of metamaterials has been of great importance since the very early stages of research in this field. When micro-structural effects play a relevant role at the macro-scale, as in the case of metamaterials, efficient modelling becomes a particularly challenging task, which still attracts the interest of several researchers. Indeed, owing to large difference of scales involved, direct numerical simulations based, for example, on the finite element method may become prohibitive, requiring alternative methods. Among others, analytical homogenization techniques, computational homogenization methods and continualization methods were proposed for this purpose.

Examples of analytical homogenization techniques are averaging techniques [49–54] built on the pivotal volume averaging technique by Willis [55], as well as asymptotic techniques, which consist of computing relevant microscopic fields and deriving macroscopic fields and effective constitutive properties by suitable volume averages over a unit cell [56–60].

Similarly, computational homogenization methods involve the formulation of two nested boundary value problems at the macro-scale and at the micro-scale [61–64] and are ideally suitable for capturing the transient response, considering finite macroscopic domains with arbitrary boundary conditions, as well as complex micro-structure geometries. Applications of computational homogenization methods revealed that, in the frequency range of excitation where locally resonant metamaterials exhibit exotic properties, the characteristic wavelengths associated with the heterogeneities can be comparable with the sizes of the micro-structural constituents of the heterogeneities, implying that inertia effects at the micro-scale cannot be neglected and shall be reflected in the local macroscopic response [61–64]. Based on this observation, homogenized constitutive relations for the macroscopic stress were obtained in Sridhar *et al.* [63], which depend on additional kinematic degrees of freedom representing the internal dynamics of a representative volume element at the micro-scale and enriching the macroscopic continuum with micro-inertia effects in a micro-morphic sense. Further developments of this approach led to the formulation of a reduced-order macroscopic homogenized continuum whose governing equations involve no additional variables to describe the micro-scale dynamics [65]. Other computational homogenization methods conceived for elastic metamaterials can be found in Roca *et al*. [66,67].

Continualization methods deliver equivalent continua starting from a Lagrangian discrete system representation. Examples are the standard methods [68–74], the standard energy-based methods [70,72,75–78], the improved and enhanced methods [73,74,79–84] and the improved energy-based methods [85–89]. Along this research vein, in the theme issue, Del Toro *et al*. [90] propose a high-frequency continualization scheme to study a class of quasi-periodic metamaterials created through the repeated arrangement of an elementary cell in a fixed direction. The elementary cell consists of two building blocks made of elastic materials and arranged according to the generalized Fibonacci sequence, giving rise to a quasi-periodic finite micro-structure. By exploiting the transfer matrix method, the frequency band structure of selected periodic approximants associated with the Fibonacci superlattice, i.e. the layered quasi-periodic metamaterial, is determined. The frequency band structures obtained from the continualization scheme are compared with those derived from the Bloch-Floquet theory to validate the proposed scheme.

When the focus of the analysis concerns the low-frequency dynamics of metamaterials, effective medium approaches can be used to provide closed-form expressions for the effective mass density, and elastic parameters of resonant and non-resonant media [91]. The effective medium approach becomes particularly suitable when the case studies concern elastic waveguides (rods, plates, half-space) decorated with an array of resonant elements, e.g. pillars, beams, ribs or discrete-like oscillators, to form a so-called elastic metasurface. For such configuration, an equivalent continuous description of the metasurfaces can be obtained by treating the resonant elements as boundary terms in the equilibrium equation of the waveguide. This approach enables to obtain compact, closed-form expressions for the dispersion law of the elastic metasurfaces, as shown in several recent research works [92–94]. Following this research approach, in the theme issue, Zeighami *et al*. [95] investigate elastic metasurfaces embedding mechanical oscillators to control a particular type of surface waves, the Scholte-Stoneley waves (SSWs), which propagate at the planar interface between a solid medium and a fluid. Two scenarios are considered, a fluid layer or a fluid half-space overlaying a solid half-space; in both cases, the elastic metasurface is at the fluid-solid interface and is endowed with a periodic array of mass-spring resonators. Analytical dispersion relations for SSWs are derived via an effective medium approach and validated via finite element simulations. The results disclose the capabilities of the elastic metasurface in filtering, trapping and converting SSWs along the fluid-solid interface. Specifically, it is observed that graded metasurfaces with decreasing resonant frequencies induce localization of SSWs at the fluid-solid interface, while those with increasing resonant frequencies induce SSW-to-shear conversion and SSW leakage as a bulk wave.

Modelling metamaterials, and composite materials more generally, is particularly challenging in the nonlinear mechanical regime and when failure occurs. In fact, nonlinear phenomena at the micro-structural level, such as fracture, decohesion, matrix cavitation and instabilities, can significantly impact the macroscopic behaviour of these materials. To tackle these complexities without resorting to computationally demanding simulations, researchers developed reliable modelling strategies based on nonlinear homogenization and multi-scale techniques within a finite deformation framework. These approaches proved effective in investigating instability phenomena and the effects of constitutive and geometrical nonlinearities, underscoring the importance of accurate modelling for predicting local instability in composite materials. Within this context, in the theme issue, Greco *et al*. [96] present a theoretical framework based on nonlinear homogenization for characterizing the failure behaviour of periodically reinforced hyperelastic composites. The proposed approach aims at addressing reinforcement/matrix decohesion and interactions between contact mechanisms and microscopic instabilities. Debonding and unilateral contact between different phases are managed using a cohesive/contact model, which features a nonlinear interface constitutive law and an accurate contact formulation within the context of finite strain continuum mechanics. The framework is demonstrated by analysing periodically layered composites under macroscopic compressive loading along the lamination direction. Numerical results illustrate how debonding phenomena, combined with fibre micro-buckling, can influence the critical loads of the composite solid. The study also explores the sensitivity of the results obtained through the proposed contact-cohesive model at finite strain.

## 4. Engineering applications

Elastic and acoustic metamaterials lend themselves to a variety of applications. The following discussion focuses on applications for controlling wave propagation in elastodynamics and acoustics.

The most common metamaterial designs rely on a periodic architecture. Periodic materials or structures are typically referred to as phononic materials or phononic structures. The concept of ‘phonon’, formally defined as a quantum of vibrational energy in an elastic medium, originated from studies on vibrations in crystal lattices. This concept is now broadly extended to refer to an eigenmode of vibration in the context of wave-like vibrations and acoustics of periodic media, establishing a connection between phonon physics and the dynamics of periodic materials and structures [1]. In the last three decades, phononic metamaterials formed by tessellation of unit cells of various shapes, geometries and material phases were proposed for wave control, including solids, liquids and gases [97,98].

In parallel, another archetype of metamaterial for wave control is the so-called locally resonant metamaterial, whose first example consists of a three-dimensional array of cubic cells, each containing a lead sphere coated with a layer of silicone rubber within an epoxy matrix [99]. Today, a locally resonant metamaterial is defined as a metamaterial with local resonance induced by appropriate resonant units. Local resonance gives rise to exotic effective properties, such as negative elastic modulus and density at certain frequency ranges. For example, in a periodic composite material consisting of soft rubber spheres suspended in water, the effective bulk modulus and density become negative at frequency ranges close to the local resonance, and both can be simultaneously negative in a narrow frequency range [100]. Similarly, negative effective elastic modulus [101–104], negative mass density [105–109] or both [110–113] can be achieved in solid elastic metamaterials. Double negativity supports further exotic dynamic behaviour, as negative refraction.

A very appealing feature of elastic metamaterials for wave conditioning is their ability to possess frequency band gaps, i.e. frequency ranges where waves do not propagate [114]. The formation mechanisms of a band gap are associated with Bragg scattering [115], local resonance [116–121] or the combination of the two [122–125]. Bragg scattering arises from the periodicity of the unit cells constituting the metamaterial structure [114] and can be explained by multiple reflections that waves experience when propagating through an inhomogeneous medium. In a periodic medium, the opening frequency of a band gap owing to Bragg scattering is determined by the relation between the wavelength of the propagating wave and the spacing between unit cells. The smaller the wavelength, the higher the frequency of the wave; hence, a smaller spacing between unit cells generally implies a higher opening frequency of the band gap. Local resonance, on the other hand, arises from the resonance of the unit cell and may appear in both periodic and non-periodic arrangements of the cells [126]. The opening frequency of a local resonance band gap occurs in proximity to the resonant frequency of the single resonator. As a result, local resonance can induce band gaps at relatively low frequencies, even if the spacing between unit cells is small, i.e. at the so-called sub-wavelength scale, with obvious advantages for the realization of compact wave conditioning devices. In recent years, a plethora of designs was proposed to realize locally resonant elastic waveguides, including beams and plates coupled with periodic arrays of small resonators [127–148]. Remarkable properties were also found in locally resonant lattice metamaterials [149,150]. For instance, the study in Bigoni *et al*. [149] introduced a lattice metamaterial structure endowed with inertial resonators, each consisting of an inclusion coated by a structural interface made of beams inclined at given angle; as a result, the lattice metamaterial exhibits tunable low-frequency stop bands, associated with localized rotational modes, to be used in the design of filtering, reflecting and focusing devices. An emerging perspective to enhance the performances of locally resonant metamaterials is to use nonlinearities, e.g. for wideband attenuation [151–153]. To this aim, the study in Shen & Lacarbonara [152] proposed lattice metamaterials with membrane-shaped resonators supporting central masses, while Zhao *et al*. [153] envisaged beams equipped with periodically distributed inertia amplifiers, whose geometric nonlinearity induces amplitude-dependent nonlinear damping effects.

In several applications at either macro- or micro-scales, the control or mitigation of surface waves play a major role. This is the context where elastic metasurfaces are particularly appealing. Indeed, thanks to local resonance, elastic metasurfaces can be designed at the sub-wavelength scale, with applications encompassing sensing and energy harvesting [154], sound absorption [155,156], non-destructive evaluation [157]; spatial modulation of the resonant frequencies in elastic metasurfaces can induce wave focusing [158,159], rainbow trapping [160–162], mode conversion [163,164]. Moreover, elastic metasurfaces can be devised for seismic isolation and vibration mitigation [92,93,165–168] at the geophysical scale. In this framework, two contributions are included in the theme issue regarding the design of periodic wave barriers (PWBs), which are large-scale phononic structures intended to mitigate ground vibrations from various sources. In the contribution by Ni *et al*. [169], PWBs are designed and analysed to mitigate ambient vibrations induced by moving loads. In particular, the authors investigate the effect of speed and frequency of moving loads on surface waves to enhance the design methodology of PWBs for reducing and isolating ambient vibrations. Two types of PWBs—periodic empty trench barriers and periodic pile barriers—are explored. The study begins by deriving a theoretical expression for the primary frequency band of surface waves propagating in an elastic half-space owing to a moving load. Comparisons with numerical results for three different types of traffic loads are conducted, showing good agreement. Additionally, experimental studies are carried out to validate the theoretical expression and numerical findings, revealing inherent properties of wave propagation caused by a moving load in an elastic half-space. Overall, when the attenuation zones of a PWB match the target frequency bands given by the theoretical expression, good vibration mitigation can be achieved. The second contribution, by Wu *et al*. [170], examines the attenuation of surface waves using periodic in-filled trench barriers in unsaturated soil. The authors define the dispersion relations of a periodic structure for surface waves in unsaturated soil and discuss the attenuation mechanism of evanescent surface waves. Subsequently, a comprehensive investigation to highlight the effects of several key parameters of unsaturated soil on the attenuation zones of the periodic in-filled trench barriers is conducted. Through this analysis, it is found that, for certain material parameters, the surface waves are attenuated across the entire frequency range owing to the viscosity of the fluid. Based on these findings, a periodic in-filled trench barrier is designed following a field test of ground vibration induced by a train, and its performance in mitigating surface waves propagating in unsaturated and saturated soils is assessed and compared through analysis in the time domain.

Besides band gaps, another important feature of elastic metamaterials for wave conditioning is wave steering. For example, building on transformation optics, elastic lenses can be designed to reroute and/or focus the propagation of elastic waves in plates or half-spaces [171,172]. In addition, wave steering can be achieved by relying on the so-called generalized Snell’s law (GSL). The GSL consists of introducing a phase gradient at the interface of two media to shape non-standard reflection and/or refraction waves. In elastodynamics, the phase shift is related to the variation of phase velocities, which can be achieved by adjusting the dynamic behaviour of locally resonant units [173–176]. In principle, covering the whole phase range may allow metamaterials to arbitrarily guide elastic waves [177,178] and, exploiting this mechanism, numerous metasurfaces were designed to guide flexural or in-plane waves in thin plates [179–183], as well as surface waves along the edge of half-spaces [184]. In the theme issue, the paper by Cui *et al*. [185] proposes a wave barrier consisting of a cluster of resonators arranged in circular shape around the vibration source in a semi-infinite medium. The authors demonstrate that keeping all resonators identical and varying their mass uniformly ensures a phase shift up to 2 π ; considering all resonators located in the same radial direction as a group, the resonator mass is assigned group by group according to the GSL, realizing directional refraction and wave energy focusing, with practical applications for isolating ground-borne vibrations and energy harvesting. These conclusions are corroborated numerically via a multiple-scattering formulation modelling the dynamic behaviour of the half-space coupled to the metabarrier.

As elastic metamaterials are especially suitable for structural/mechanical vibration mitigation, acoustic metamaterials are ideal candidates for noise insulation. In the typical frequency range of acoustic waves, band gaps owing to local resonance were obtained by membrane-like resonance absorbers [186–190] and various types of internal resonators [191–195]. Moreover, three-dimensional phononic crystals specifically tailored for acoustic applications were devised [196–198]. Interesting reviews on potential uses of phononic crystals in acoustics can be found in Gorishnyy *et al.*, Maldovan and Chen *et al*. [199–201], including acoustic isolation, noise suppression, acoustic waveguides, acoustic super-lenses, negative refraction, acoustic cloaking and vibro-acoustic energy harvesting. Tunable metamaterials for acoustic applications were also proposed [202,203]. For example, the study by Ning *et al.* [202] proposed a metamaterial consisting of an aluminium frame structure including resonant elements, each made of a neoprene airbag and a tungsten balancing mass, which can be tuned by varying gauge pressure or gas temperature in the airbag to attain low-frequency band gaps [202]; in Wen *et al*. [203], a metamaterial was devised consisting of a waveguide coupled with Helmholtz resonators, each incorporating an accordion origami as side cavity, the volume of which can be adjusted by pneumatic devices. In the theme issue, Hermann *et al*. [204] present the design and experimental validation of a three-dimensional printed labyrinthine metamaterial for vibro-acoustic applications. The unit cell is designed with a labyrinth pattern and optimized based on Bloch-Floquet analysis coupled with finite element analysis in Comsol Multiphysics, aiming to maximize the band gap. Upon optimization, finite-size sandwich specimens are realized by including the metamaterial between top and bottom plates. Three different sets of specimens are considered, using polymethyl methacrylate (PMMA) for both cells and plates, PMMA for cells and gypsum for plates, a photopolymer for both cells and plates. The metamaterial’s properties are analysed from a purely mechanical and a vibro-acoustic (i.e. considering solid-air interactions) point of view, assuming the transfer function and the sound transmission loss at normal incidence as evaluation metrics. Mechanical vibro-impact experimental tests are in good agreement with predictions from numerical models, confirming the existence of a large band gap in the low- to mid-frequency range. Building on these results, additional numerical investigations unveil the strong dependence between the metamaterial’s vibro-acoustic performances and the connections at the interface between metamaterial and plates, concluding that, by appropriate design of the interface, vibro-acoustic performances largely exceeding benchmark performances in the field can be obtained by the proposed concept.

## Data Availability

This article has no additional data.
